# The usefulness of liquid-based cytology for endoscopic ultrasound-guided tissue acquisition of solid pancreatic masses

**DOI:** 10.3389/fmed.2022.943792

**Published:** 2022-08-16

**Authors:** Masahiro Itonaga, Reiko Ashida, Masayuki Kitano

**Affiliations:** Second Department of Internal Medicine, Wakayama Medical University, Wakayama, Japan

**Keywords:** liquid-based cytology (LBC), endoscopic ultrasound-guided tissue acquisition (EUS-TA), pancreatic masses, smear cytology (SC), next-generation sequencing (NGS), endoscopic ultrasound-guided fine needle aspiration (EUS-FNA)

## Abstract

Liquid-based cytology (LBC) is used primarily for cervical cytology, although it is also used for analyzing liquid samples such as urine and ascites specimens, as well as fine needle aspiration material, such as those obtained from breast and thyroid. The usefulness of the LBC method for endoscopic ultrasound-guided tissue acquisition (EUS-TA) of solid pancreatic masses was recently reported. The LBC method can produce multiple pathological slides and can be applied to immunocytochemistry and genetic analyses. In this article, we review the usefulness of LBC for EUS-TA of solid pancreatic masses.

## Introduction

Endoscopic ultrasound-guided tissue acquisition (EUS-TA) is widely used for the pathological diagnosis of intra-abdominal masses, especially pancreatic masses (1, 2). Cytological diagnosis in EUS-TA is generally performed by smear cytology (SC), in which the collected specimen is smeared directly on a glass slide. This method is inexpensive, easy to use, and available at most institutions (3). The sensitivity of EUS-TA using SC for the diagnosis of pancreatic masses is 64–94% (4). However, SC is very sensitive to insufficient cell counts, smears filled with inflammatory cells or blood cells, drying artifacts, crushing artifacts, or thick tissue fragments, which can mask cytological features and result in suboptimal diagnosis (5).

In liquid-based cytology (LBC), collected cells are suspended in a liquid, homogenized, and smeared on a glass slide, and it has attracted attention as an alternative method to prevent blood contamination and cell drying/depletion, which are drawbacks of SC (6). LBC is primarily used for cervical cytology (7), although its diagnostic efficacy in the analysis of non-gynecological samples such as fine needle aspiration specimens of the breast, thyroid gland, and lymph nodes was recently reported (8, 9). However, LBC is not commonly used for pancreatic specimens obtained by EUS-TA, and whether the diagnostic accuracy of LBC is superior to that of SC remains controversial (5, 6, 10–13).

Liquid-based cytology specimens are not only useful for cytological diagnosis, but are also valuable for obtaining genetic information to guide diagnosis, prognosis, and treatment. The advantages of genetic analysis using LBC specimens include ease of handling, storage, and transportation, and the test is not burdensome for the patient because samples can be collected during routine examinations (14). Akahane et al. reported that the quality of the genomic DNA for next-generation sequencing (NGS) is better preserved in LBC samples than in formalin-fixed paraffin embedded (FFPE) tissues even after several years of storage (15). Several recent reports have described genetic analysis using LBC specimens obtained by EUS-TA (14, 16, 17).

In this article, we review the usefulness of LBC for EUS-TA of solid pancreatic masses.

## Types and principles of liquid-based cytology

Liquid-based cytology can be broadly classified into two methods according to the composition of the fixation and preservation solution and the specimen preparation technique: ThinPrep (Hologic Inc., Marlborough, MA, United States) and SurePath (BD Diagnostics, Burlington, NC, United States). The ThinPrep method uses a ThinPrep 5,000 processor to gently disperse the cell suspension and homogenize the cell population. The cells are then automatically collected on disposable polycarbonate filters and transferred to glass slides within a 20 mm diameter circle (18). In the SurePath method, centrifugation is used to attach the cell pellets to the glass slides by gravitational sedimentation and electrical adhesion (18). There are differences in cell morphology, background, and artifacts between the ThinPrep and SurePath methods (19, 20). The specimens prepared by the ThinPrep method have a clean background and are characterized by large cell clumps; the nuclei are larger than those prepared by the SurePath method. In the ThinPrep method, the number of cells on the glass slide is reduced by the presence of inflammation, blood, and mucus. By contrast, specimens prepared by the SurePath method are characterized by scattered single cells and bare nuclei, as well as the presence of leukocytes in the background. In the SurePath method, a three-dimensional architecture, large cell masses, and overlapping nuclei are also observed.

## Advantages and disadvantages of liquid-based cytology

The advantages of LBC over SC include (1) specimen uniformity, (2) cell retrieval, (3) reduced burden on the cytologist, and (4) specimen diversity (21, 22).

### Specimen uniformity

**FIGURE 1:**
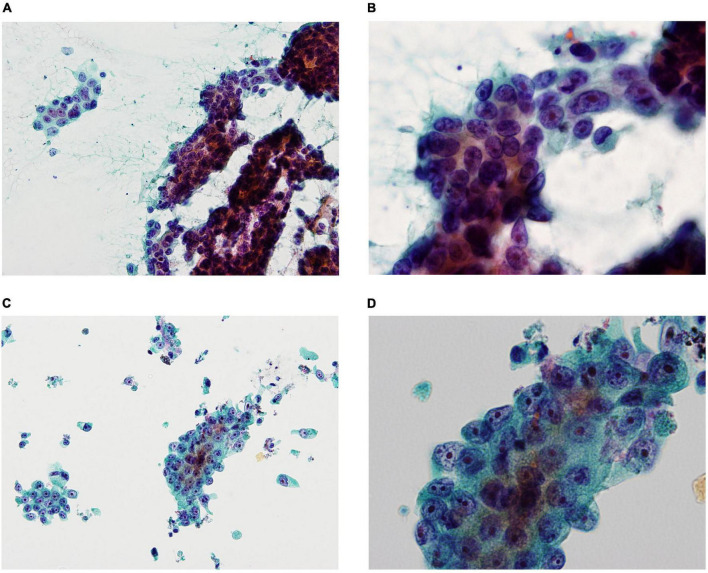
Smear cytology (SC) and liquid-based cytology (LBC) images demonstrating pancreatic ductal adenocarcinoma (**A:** SC ×200, **B:** SC ×600, **C:** LBC ×200, **D:** LBC ×600). SC was frequently contaminated by blood and mucus contaminations, obscuring evaluation of the cellular elements **(A)**, whereas LBC reduced blood and mucus contaminations, resulting in clean backgrounds **(C)**. SC showed more cell overlap compared to LBC **(B,D)**.

Liquid-based cytology reduces the number of inadequate specimens by removing blood and mucus, as well as that of specimens with poor fixation due to drying. In addition, specimens are more uniform and show less cell overlap, and specimen processing can be standardized for cell collection and preparation (Figure 1).

### Cell collection performance

Cell collection performance is improved because the presence of cell remnants on the collection device and cell detachment are reduced.

### Reduced burden on the cytologist

The diagnostic time is decreased by reducing the number of fields of view, and tumor cells can be easily identified by decreasing the number of contaminants.

### Specimen diversity

Multiple specimen preparation is possible, and samples can be used for immunostaining and genetic analysis.

## Disadvantages of liquid-based cytology

The disadvantages of LBC over SC include (1) cost and (2) requirement for training in cellular analysis, as well as variability in judgment and diagnostic criteria (22).

### Cost

Liquid-based cytology requires a high initial investment and consumable costs. The high initial investment and maintenance costs associated with the ThinPrep have limited its use to large institutions, making it difficult to use in small institutions. However, SurePath requires only a centrifuge and inexpensive consumables, and it is thus feasible for small institutions. Although LBC is more expensive than SC, the reduction in inadequate smears alone would make LBC less expensive in the long run compared with CS (23). Further studies regarding the cost-effectiveness of LBC would be helpful to determine the applicability of LBC in resource-limited settings.

### Training

The LBC method requires special training and practice in reading the results and making a diagnosis because of cell swelling and shrinkage, discrepancies in the aggregates, and loss of background information that might provide a basis for diagnosis. Training is required for reading cellular findings based on specific diagnostic criteria.

## The specimen processing for liquid-based cytology

**FIGURE 2:**
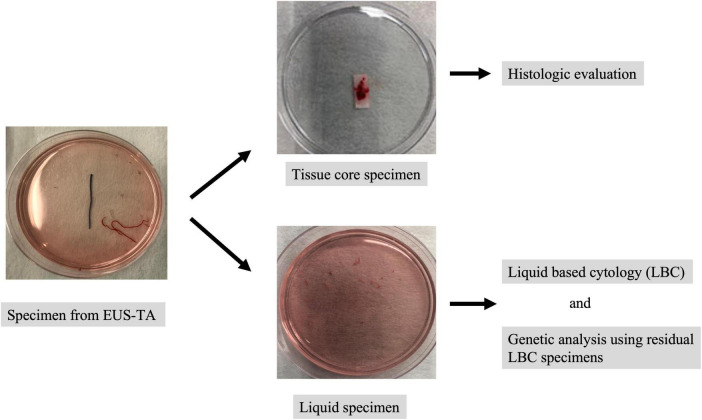
The specimen processing for liquid-based cytology (LBC). The specimens corrected by EUS-TA were immediately suspended in preservative fluid. After extracting tissue core specimens for histological analysis, liquid specimens were collected for LBC analysis. The residual LBC specimens were stored at 4°C until DNA extraction for genetic analysis.

The specimens corrected by EUS-TA were immediately suspended in preservative fluid (CytoRich Red, Thermo Scientific, Waltham, MA, United States). After extracting tissue core specimens for histological analysis, liquid specimens were collected for LBC analysis (Figure 2). In the ThinPrep methods, cells were isolated from the fluid by vacuum filtration and were transferred to the slide using air pressure for adherence (10). In the SurePath method, after centrifugation, purified water was added to the sample, which was dropped and smeared onto a BD SurePath PreCoat slide (Becton Dickinson Japan) with chambers 13 mm in diameter (11). Slides for LBC were prepared and fixed in 95% ethanol for 24–48 h. These slides were stained using the Papanicolaou procedure and examined under light microscopy. The residual LBC specimens were stored at 4°C until DNA extraction for genetic analysis.

## Diagnostic performance of liquid-based cytology for endoscopic ultrasound-guided tissue acquisition of solid pancreatic masses

### Comparison of liquid-based cytology and smear cytology

There are ten studies in the literature comparing LBC and SC for EUS-TA of solid pancreatic masses (Table 1). Chun et al. reported the results of a randomized single-center study comparing SC without rapid on-site evaluation (ROSE) with LBC for EUS-TA of solid pancreatic masses (5). In this randomized study (5), inadequate samples and bloody backgrounds were significantly less frequent in the LBC group than in the SC group (LBC, 1.78% vs. SC, 5.33%, *P* = 0.015; LBC, 1.8% vs. SC, 85.2%, *P* < 0.001, respectively), whereas the diagnostic accuracy did not differ significantly between the two groups (LBC, 88.0% vs. SC, 83.8%; *P* = 0.276). Qin et al. also reported the diagnostic accuracy was comparable between the two groups (24). On the other hand, three studies reported the sensitivity was significantly higher in the SC group than in the LBC group (25–27), while another three studies reported the sensitivity was significantly higher in the LBC group than in the SC group (3, 6, 11). A meta-analysis comparing LBC and SC without ROSE for EUS-TA of solid pancreatic masses showed that the sensitivity was significantly higher in the LBC group than in the SC group (LBC, 76% vs. SC, 68%; *P* < 0.05) (28). Thus, although the superiority of LBC over SC for the detection of pancreatic solid masses by EUS-TA is controversial, LBC may replace SC in facilities in which ROSE is not available.

**TABLE 1:** Comparison of the diagnostic performance of LBC and SC in EUS-TA of solid pancreatic masses.

References	Year	Study design	Sample size	LBC technique	ROSE available	Diagnostic performance		Outcome
						Sensitivity (LBC vs. SC) (%)	Specificity (LBC vs. SC) (%)	
de Luna et al. (30)	2004	Retrospective	67	ThinPrep	Yes	58 vs. 77	100 vs. 100	LBC < SC
LeBlanc et al. (25)	2010	Prospective	50	ThinPrep	Yes	62 vs. 98	100 vs. 100	LBC < SC
Lee et al. (26)	2011	Prospective	58	ThinPrep	No	75 vs. 93	100 vs. 100	LBC < SC
Qin et al. (24)	2014	Prospective	72	ThinPrep	No	73 vs. 70	100 vs. 100	LBC = SC
van Riet et al. (3)	2016	Prospective	71	ThinPrep	No	80 vs. 63	100 vs. 100	LBC > SC
Hashimoto et al. (11)	2017	Retrospective	126	ThinPrep and SurePath	No	90 vs. 64	100 vs. 100	LBC > SC
Yeon et al. (27)	2018	Prospective	75	SurePath	No	61 vs. 86	100 vs. 100	LBC < SC
Zhou et al. (6)	2020	Retrospective	514	SurePath	No	70 vs. 54	100 vs. 99	LBC > SC
Chun et al. (5)	2020	Randomized controlled	170	SurePath	No	88 vs. 83	100 vs. 100	LBC = SC
Huang et al. (31)	2021	Retrospective	52	ThinPrep	Yes	87 vs. 96	100 vs. 100	LBC = SC

ROSE, rapid on-site evaluation; LBC, liquid-based cytology; SC, smear cytology.

Zhang et al. performed a meta-analysis comparing LBC and SC with ROSE for pancreatic solid masses obtained by EUS-TA (29). The sensitivity was significantly higher in the SC group than in the LBC group (LBC, 60% vs. SC, 90%; *P* < 0.05). Although this result suggests that LBC is less useful in facilities in which ROSE is available, the studies included a small number of cases (30, 31), and large-scale studies are needed in the future.

### Comparison of liquid-based cytology alone, smear cytology alone, and liquid-based cytology combined with smear cytology

Four studies compared LBC alone, SC alone, and LBC combined with SC for EUS-TA of solid pancreatic masses (Table 2). Zhou et al. reported that the diagnostic accuracy was significantly higher in the LBC combined with SC group than in the LBC alone group (LBC combined with SC, 86.5%; LBC, 76.1%; *P* < 0.001) (6). Work from our group showed that the diagnostic accuracy was significantly higher in the LBC combined with SC group than in the SC group (LBC combined with SC, 94.1%; SC, 69.6%; *P* < 0.001) (10). A meta-analysis comparing SC, LBC, and LBC combined with SC reported that the pooled sensitivity was significantly higher in the LBC combined with SC group than in the SC alone group and LBC alone group (LBC combined with SC, 87%; SC, 68%; LBC, 76%; *P* < 0.05) (28). These results suggest that LBC combined SC is superior to LBC alone or SC alone for the clinical evaluation of pancreatic lesions.

**TABLE 2:** Comparison of the diagnostic performance of LBC alone, SC alone, and LBC combined with SC in EUS-TA of solid pancreatic masses.

References	Year	Study design	Sample size	LBC technique	ROSE available	Diagnostic performance		Outcome
						Sensitivity (%)	Specificity (%)	
Lee et al. (26)	2011	Prospective	58	ThinPrep	No	93.2 vs. 97.7 (SC vs. LBC with SC)	100 vs. 100 (SC vs. LBC with SC)	SC = LBC with SC
Yeon et al. (27)	2018	Prospective	75	SurePath	No	78 vs. 81 (SC vs. LBC with SC)	100 vs. 100 (SC vs. LBC with SC)	SC = LBC with SC
Itonaga et al. (17)	2019	Retrospective	204	ThinPrep	No	67 vs. 93.2 (SC vs. LBC with SC)	90 vs. 100 (SC vs. LBC with SC)	SC < LBC with SC
Zhou et al. (6)	2020	Retrospective	514	SurePath	No	71 vs. 83.9 (LBC vs. LBC with SC)	100 vs. 99 (LBC vs. LBC with SC)	LBC < LBC with SC

ROSE, rapid on-site evaluation; LBC, liquid-based cytology; SC, smear cytology.

### The diagnostic performance of liquid-based cytology related to endoscopic ultrasound-guided tissue acquisition needle type

In the above literature on the diagnostic performance of LBC, EUS-guided fine needle aspiration (EUS-FNA) needles were used for EUS-TA of solid pancreatic masses. New EUS-TA with new EUS-guided fine needle biopsy (FNB) needles was demonstrated to outperform EUS-TA with EUS-FNA (32, 33). Moreover, ROSE is also possible using these FNB needles (34). Therefore, it is likely that EUS-FNB needles will replace EUS-FNA needles in the near future, and subsequently, cytology will be replaced by histology. Tomita et al. reported that the diagnostic accuracy of LBC with a 25-gauge FNA needle and histology with a 22-gauge FNB needle for solid pancreatic lesions were comparable (35). Future large-scale studies comparing LBC (with or without ROSE) with a EUS-FNA needle and histology with a EUS-FNB needle are needed.

## Immunocytochemistry using liquid-based cytology specimens

Liquid-based cytology specimens allow for multiple specimen preparations and immunostaining of cytology specimens. Rossi et al. reported that primary pancreatic malignant lymphoma was successfully diagnosed using immunostaining of LBC specimens for EUS-TA (36). Son et al. showed that immunostaining for TTF-1 and CD56 could be used to diagnose metastatic pancreatic cancer using LBC specimens from EUS-TA (37). Thus, in cases in which immunostaining is difficult because of insufficient tissue samples, a detailed diagnosis can be made by immunostaining LBC specimens. However, the usefulness of immunostaining using LBC specimens has only been shown in case reports, and studies using a large number of patients are needed.

## Genetic analysis using residual liquid-based cytology specimens

Because estimating tumor fraction in NGS samples is critical for annotating the results, criteria for the preparation and storage of FFPE tissues for cancer molecular testing, particularly NGS analyses, were proposed in Japan (38). However, genomic DNA in FFPE tissues is degraded over time, and FFPE tissues need to be used within 3 years for NGS analyses (38). By contrast, the DNA is preserved in LBC specimens, and the DNA quality for NGS is maintained even after 5 years of storage compared with FFPE tissues (15). In addition, residual LBC specimens can be directly used for DNA extraction without any additional procedures such as FFPE preparation (39). Therefore, residual LBC specimens could serve as an alternative source of material for molecular testing in the diagnosis of cancer.

Genetic analysis using residual LBC specimens obtained by EUS-TA in pancreatic masses was performed in three studies in the literature (Table 3). Sekita-Hatakeyama et al. reported that the use of residual LBC specimens for *K-ras* mutation analysis improved the diagnostic accuracy of EUS-TA (16). In this study, the combined use of the results of Cellblock (CB) and *K-ras* mutation analyses increased the sensitivity and accuracy of the diagnosis of PDAC (90.3 and 90.7%, respectively) as compared to that achieved with CB diagnosis alone (77.4 and 81.3%, respectively). We reported that *K-ras* mutation analysis was successful in 98.6% (274/278 patients) of residual LBC specimens, and *K-ras* gene status predicted the therapeutic responses to gemcitabine and nab-paclitaxel therapy, as well prognosis, in unresectable pancreatic ductal adenocarcinoma patients (17). In this study, patients with the wild-type gene showed significantly longer progression-free survival and overall survival than patients with mutant Kras [6.9/5.3 months (*P* = 0.044) vs. 19.9/11.8 months (*P* = 0.037), respectively]. Sekita-Hatakeyama et al. reported that NGS analysis targeting six genes was successful in 84.6% of patients (44/52), and the NGS analysis using LBC specimens was reliable and could support a morphological diagnosis (14). In this study, the analysis identified 54.5% of PDAC patients carrying KRAS and CDKN2A/PIK3CA/TP53/SMAD4 mutations, whereas 91% of benign patients showed no mutations. Although these studies suggest the efficacy of genetic analysis using LBC specimens obtained by EUS-TA for pancreatic masses, a small number of genes were analyzed. Further studies with a large number of genes are needed.

**TABLE 3:** Genetic analysis using residual LBC specimens in EUS-TA of solid pancreatic masses.

References	Year	Study design	Sample size	Gene	Main findings
Sekita-Hatakeyama et al. (16)	2018	Retrospective	81	*K-ras*	*K-ras* mutation analysis using residual LBC samples was successful in all patients. *K-ras* mutation analysis using residual LBC specimens improves the diagnostic accuracy of EUS-TA.
Itonaga et al. (17)	2022	Retrospective	278	*K-ras*	*K-ras* mutation analysis was successful in 98.6% of residual LBC specimens and the analysis of the *K-ras* gene status could be used to predict therapeutic responses to GA therapy and prognosis in unresectable PDAC.
Sekita-Hatakeyama et al. (14)	2022	Retrospective	52	6 genes	Gene analysis targeting six genes was successful in 84.6% of patients. The analysis identified 54.5% of PDAC patients carrying KRAS and CDKN2A/PIK3CA/TP53/SMAD4 mutations, whereas 91% of benign patients showed no mutations.

LBC, liquid-based cytology; GA, gemcitabine and nab-paclitaxel; PDAC, pancreatic adenocarcinoma.

## Conclusion

The LBC method provides uniform specimens and shows a high cell collection efficiency. Its diagnostic performance in EUS-TA for pancreatic masses is expected to be good, and it can be used for immunohistochemical and genetic analyses using residual LBC specimens.

## Author contributions

MI, RA, and MK designed this review and drafted the manuscript. MI and MK wrote the manuscript. All authors contributed to the article and approved the submitted version.
